# Patients’ perspectives on point-of-care diagnostics and treatment by emergency medical technicians in acute COPD exacerbations: A qualitative study

**DOI:** 10.1186/s13049-022-00999-2

**Published:** 2022-02-19

**Authors:** H. M. Christensen, P. I. Pietersen, C. B. Laursen, D. Wittrock, G. Nadim, G. Jørgensen, L. B. Nielsen, M. K. Sørensen, I. L. Titlestad, A. T. Lassen, S. Mikkelsen

**Affiliations:** 1 Department of Respiratory Medicine, Odense University Hospital, Sdr. Boulevard 29 entrance 87-88, 5000 Odense C, Denmark; 2 Department of Clinical Research, University of Southern Denmark, Odense, Denmark; 3 Ambulance Syd, Odense, Denmark; 4 Emergency Medicine Research Unit, Odense University Hospital, Odense, Denmark; 5 Department of Health Planning, Prehospital Services, Region of Southern Denmark, Vejle, Denmark; 6 Responce & Falck Danmark, Kolding, Denmark; 7 The Prehospital Research Unit, Region of Southern Denmark, Odense University Hospital, Odense, Denmark

**Keywords:** Patient perspectives, COPD, Exacerbation, Prehospital, Point-of-care

## Abstract

**Background:**

In Denmark emergency medical technicians transport patients with acute COPD exacerbations to the nearest emergency department. From a clinical and economic perspective, this transport and assessment at the hospital may be inconvenient if the patient is immediately discharged from the emergency department. We established an emergency technical technicians point-of-care diagnostics and treatment program of patients with COPD with use of ultrasound and blood analysis. Patients' perspectives on treatment at home and sense of security are important to qualify clinical practice at home with patients with acute exacerbation.

**Aim and objectives:**

To explore patient's and relatives' experience of treatment at home during emergency calls due to COPD in exacerbation and to investigate their attitude to avoid hospitalization as well as experience of stress during point-of-care diagnostics in their own home.

**Method:**

A qualitative study comprising semi-structured interviews with 16 patients carried out from April 1st, 2019 to March 31st, 2020 in Denmark. Data was analysed inspired by Malteruds’ text condensation and informed by Critical Psychology with first person perspective focusing on the patient's views on point-of-care diagnostics and treatment of their COPD in acute exacerbation.

**Results:**

The interviews revealed that in order to ensure an experience of quality in the assessment and treatment of patients in their own homes, it was important that the ambulance staff showed great safety and experience in the use of the technical equipment and treatment of dyspnea. It was also of importance that the patients felt confident that their general practitioner followed up on the home treatment initiated.

**Conclusion:**

Patients’ perspectives showed that point-of-care diagnostics and treatment of acute COPD in exacerbation was considered a qualitative offer by the patients and their relatives. At the same time, it was crucial that the emergency medical technicians showed experience and safety in handling shortness of breath as well as the technical equipment.

*Trial registration*: Approved by the Danish Data Protection Agency Project-ID: 20/24845.

## Introduction

Chronic obstructive pulmonary disease (COPD) is considered one of the leading causes of mortality worldwide [1, 2]. In Denmark, there are approximately 270.000 persons with clinical severe COPD GOLD stage B-D [2, 3], and approximately 20.000 are admitted to hospitals in Denmark each year due to COPD exacerbations [4]. A COPD exacerbation is characterised by acute worsening of symptoms such as breathlessness, increased sputum, and cough [5]. Exacerbations contribute to the progressive course of COPD, with consequences such as reduced life quality, repeated hospital admissions, and increased health-care related costs for the patient and society.

In Denmark, emergency medical technicians (EMT) transport patients with acute COPD exacerbations to the emergency department (ED) [6]. Often the patients are treated for their exacerbation at the ED with bronchodilators, corticosteroids, and if appropriate also antibiotics [5, 7]. If other comorbidities are excluded and the patient responds to the initial treatment, the patient will typically be discharged after a short evaluation within 24 h of admission [8]. This transport and assessment at the hospital may be inconvenient for the patients’ and relatives’ life quality, wellbeing, and pose a risk for nosocomial infection. Also, from a clinical and health care economic perspective, short stays at the hospital are inconvenient. More and more people are living longer with one or more chronic diseases, leading to increased crowding of hospitals. To reduce this crowding, there is a political agenda to increase the number of patients with chronic diseases being treated at home or in an outpatient setting rather than being admitted to hospitals [9, 10]. Technological development and research in diagnosis and treatment at the "first point of contact" has a significant potential to provide new insights and support the development of new outpatient solutions for patients with chronic diseases such as COPD.

Assessment and treatment of COPD exacerbation in the patients’ home could reduce the economic cost, time to goal-directed treatment, and possibly the patient’s level of distress if hospital admissions can be avoided. A recent feasibility study from our research group assessed an organisational set-up allowing the EMTs to perform point-of-care diagnostics (lung ultrasound, blood analysis) and initiate treatment when an emergency ambulance was called to the home of a patient with possible COPD exacerbation. The set-up was found to be practically feasible and hospital admission could be avoided in nearly half of the included patients [11, 12].

Patients' perspectives on diagnosis and treatment at home and sense of security are considered important to qualify clinical practice, patients safety, and treatment efficacy [13] Likewise, in the health care system there is an increased focus on the positive impact that knowledge about the patients’ perspectives may render. Knowledge of the patients’ perspectives may thus contribute to prioritisations in daily clinical practice and optimise the quality of care delivered to the individual patients [14, 15]. The present study aimed to explore patients’ and relatives' experience of treatment at home during emergency calls due to COPD exacerbation. Furthermore, we aimed to investigate the patients’ attitude towards potentially avoidable admissions to hospitals as well as patients’ experience of stress during point-of-care diagnostics in their own homes. Patients enrolled in this study were drawn from the group of participants who had entered into a previous study [11, 12].

## Study-design and settings

**Fig. 1:**
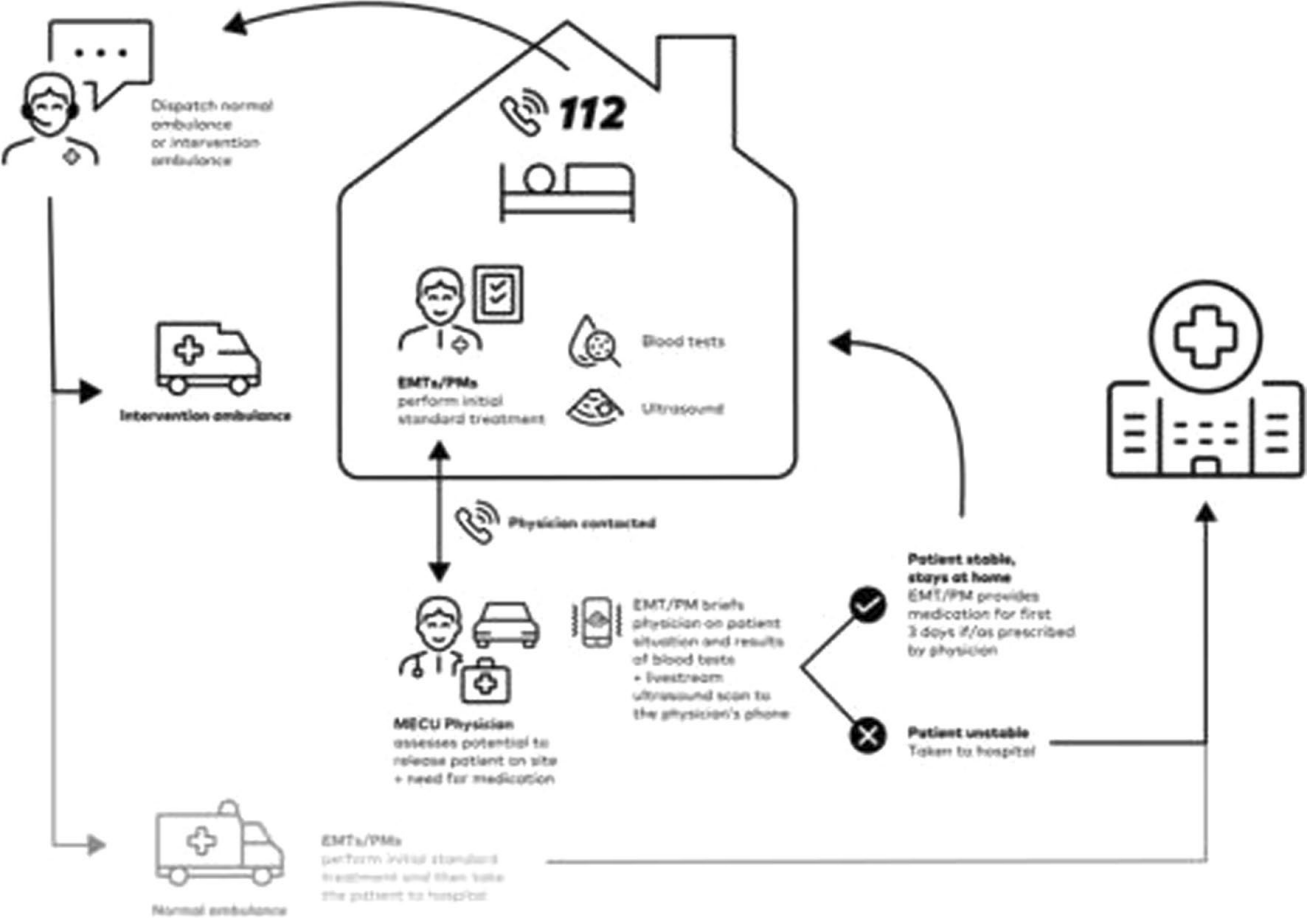
Main study overview [11]

In the basic quality assurance study, EMTs were trained to perform advanced point-of-care diagnostics (lung ultrasound, blood analysis) in patients with dyspnoea as overall complaint and previously been diagnosed with COPD. Following treatment, the EMTs were authorised to release the patient at the scene after telephone consultation with an anaesthesiologist [6, 11]. If the diagnostic findings indicated a need for treatment of the COPD exacerbation, treatment with corticosteroids and/or empirical antibiotics was initiated onsite [11]. Although an elaborate selection process resulted in very few patients being eligible for the study, almost half of the patients that were included in the study were released following treatment. This study was carried out in a prehospital setting in the Region of Southern Denmark from October 1st, 2018 to May 31st, 2019 [11, 12]. An overview of the main study is presented with permission in Fig. 1 [11].

The present sub-study was conducted as a qualitative study comprising semi-structured interviews to gain knowledge of patients’ perspectives on point of care treatment in COPD exacerbations. The interviews took place after the patient had participated in the main study and were carried out from April 1st, 2019 to March 31st, 2020.

## Design

**Fig. 2:**
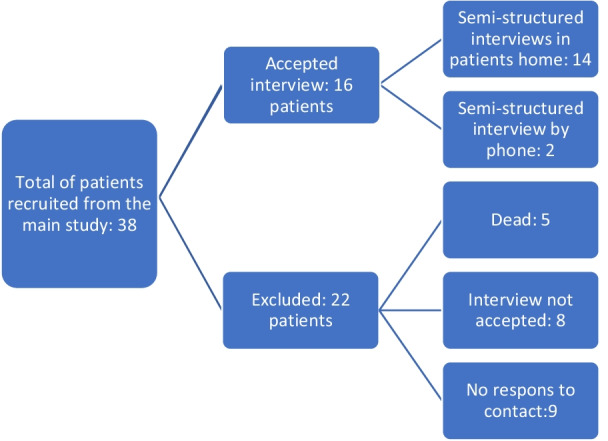
Flowchart recruitment

A preliminary meeting was held with the supporting stakeholders in the main project to determine the objectives, goals, and target group in this sub-study. A qualitative approach was chosen in the form of semi-structured interviews to gain knowledge from a first-person perspective from patients who had participated in the COPD point-of-care intervention. Patients will prospectively in this article be referred to as informants. The informants were selected consecutively from the main study reflecting differences in gender, age, and civil status. We included both informants who were admitted to the hospital after assessment at home and informants who were released in their homes following treatment. The informants were geographically scattered within the Region of Southern Denmark. The inclusion criteria were thus informants who had participated in the main research study. Exclusion criteria from the main study were the following: (1) Informant needing an interpreter. (2) Inability to understand information about the research project. An overview of the recruitment is presented in Fig. 2.

The semi-structured interviews were performed by one researcher with many years of experience in qualitative research design (HMC). The semi-structured interviews were conducted based on an interview guide inspired by Kvale and Brinkmann [16] and with a critical psychological approach to capture the first-person perspective on experiences as well as assessments of the intervention and the quality and applicability of the treatment [17, 18]. The interview guide contained open questions to enable the informants to talk about their experiences from a first-person perspective. All informants were offered the opportunity that the interviews could be conducted in their own homes. The interview guide was structured around two broad themes: (1) The informants’ conduct of everyday life with COPD and related sub-themes. (2) The informants’ experience of the EMT-led point-of-care diagnostic program related to the specific acute exacerbation of the COPD requiring the assistance of an ambulance. Attention was directed at informant’s conduct of everyday life, including issues related to health and disease, and their life circumstances. Interviews were audio recorded and transcribed verbatim.

For presentation in this paper, quotes were translated from Danish by the author with the assistance of an English language editor.

### Data analysis

The thematic analyses of interview transcripts were theoretically informed by critical psychology [17, 18], and carried out with inspiration from Malterud’s text condensation [19]. The researcher read the transcripts several times in a search for meaning units. The meaning units were identified drawing on critical psychological concepts with a focus on first-person perspectives. Critical psychology is based on historical, dialectical materialism, and views subjects as dialectically interacting with social structures in concrete action contexts. A subject does not only live under certain societal conditions, he/she also influences and produces conditions. We are historically and socially embedded as human beings, where history and society influence how possibilities and constraints in the conduct of everyday life and through life trajectories come about, and how they are experienced and acted upon. Insight into personal strategies, historicity along with social conditions and meanings from a first-person perspective are necessary to understand the ways persons think, act and relate to possibilities and constraints in specific ways [17, 18]. This approach supports knowledge within the informants’ experiences about concerns, conditions, and their meanings, along with perceived possibilities and constraints of action. Analysis of these perspectives aimed to highlight what the informants experienced as conditions and their meanings, along with the possibilities and constraints for acting in particular circumstances. The analysis focused on the informants’ views on point-of-care diagnostics and treatment of their COPD in acute exacerbation at home.

### Ethical considerations

All study procedures were carried out in accordance with the Helsinki Declaration [20]. The study was approved by the Danish Data Protection Agency [21] (Project-ID: 20/24845). Currently, the Danish National Committee on Health Research Ethics does not require the approval of studies with qualitative methods (such as participant observation and interviews). To ensure anonymity, all informants are identified by numbers only and represented by numbers in this article. Written informed consent was obtained before data collection.

## Results

Thirty-eight informants were drawn from the main study, sixteen informants accepted to participate in the semi-structured interviews. Twenty-two informants did not participate in the interviews: The predominant reasons for the patients refusing to participate were severe illness and co-morbidities resulting in other major concerns and fatigue. Five patients died before contact and eight informants did not respond to contact by the investigator. The sixteen informants who agreed to participate in the semi-structured interviews reflected both gender, different ages, and civil status, an overview is presented in Table 1. The study group comprised both informants who were admitted to the hospital after assessment at home and patients who were released at the scene following treatment. The informants were geographically scattered within the Region of Southern Denmark. Fourteen informants accepted interviews in their own homes while two informants preferred interviews by telephone. The interviews lasted from 20 to 60 min. Three informants chose to have their spouses participate with them throughout the interview. The communication between the spouses during the interviews was interacting so that one could not analyse the interviews with both spouses detached from each other. Relatives are therefore not represented separately in the results.

**Table 1:** Characteristics of informants

Characteristics	Number
Gender of informant: Male/Female	7/9
Age: Mean (range)	71,56 (55–82)
Single/not single	10/6
After point of care treatment: retained at home/hospitalised	9/7

Four main themes emerged from the data analysis. All themes were interwoven but are presented separately below:

1. The patient’s everyday life with COPD
2. The feeling of not being alone
3. The need for someone to demonstrate professional experience and assistance when falling ill
4. The patient’s usual relationship with the general practitioner (GP)

Three of the interviewed informants did not remember that they had called for emergency help, nor did they recall the point-of-care procedures. All three explained that impaired memory was common to them. They did, however, contribute to the study with their thoughts and wishes for future treatment of COPD exacerbations. There was no difference in the comments between the informants who were hospitalised after the onsite examination and treatment or not. The interview guide focused on the onsite situation, but the patients also commented on the purpose of the study, namely, to avoid hospitalisation if possible.

## The patient’s everyday life with COPD

All the informants in this study had severe COPD. When they talked about an exacerbation, they didn’t call it an exacerbation, they named it “extraordinary breathlessness”, “pneumonia” or “not being able to walk even a small distance without losing breath”. The informants expressed that exacerbations often happened suddenly without any warning. It was also a common experience that if the informants were transported to the hospital, they were often discharged from the ED after a few hours. Because of this, they rated being transported to a hospital as stressful.

*They [The EMTs] took me out to the ambulance, they did, and drove me to the emergency room and then I did not get further [into the hospital]. Then I was sent home in a taxi at night which I did not want. I do not like to be sent home at night. I do not want to be sent home at night (P2)*

*I was so glad that I was not sent to the hospital. That was a relief—yes, a relief exactly (p9)*

It was recognised by the informants that if, as an example, their oxygen levels were acceptable, they would not be allowed to stay in the hospital no matter how sick they felt. A common experience was, that if they were hospitalised for more than a few hours, the COPD symptoms decreased in a longer period after being discharged. Breathlessness was expressed by the informants as being followed by severe anxiety. The informants expressed that the longer their experience with COPD, the better their ability to cope with anxiety was. Still, it was considered as a great help/comfort to have someone nearby—not having to be alone while coping with anxiety. The informants described a search for a sense of security when having an acute exacerbation. The presence of the EMT helped which meant that the extended time during the point-of-care procedures was considered a good thing. The informants made the emergency call when it almost felt like it was too late. This was explained by their habitual living with symptoms such as dyspnoea. To the informants, living with severe dyspnoea in their everyday life was considered as a fact of life condition. Therefore, they considered it difficult to recognize when the dyspnoea had transferred into an acute exacerbation. It was also argued by the informants that because they had an experience of being discharged immediately if being transported to the hospital, they tried to cope with the symptoms as long as possible.

*Well, compared to taking me to the hospital and then five hours later to send me home again, then this is much better. The results were there immediately, and the results were the same: They could not see anything on the lungs with the scanner and things. Well, yes, they took blood samples and scanned me. I do not know if I remember it all. When I call the ambulance then it is as if I do not remember anything. In fact, it is usually almost too late when I call the ambulance (P3)*

Some of the informants experienced being in such a bad condition, that they were not quite aware of what was done at the scene by the EMT. Several of the patients also expressed not being able to remember one emergency call from another.

*Well, you know, these things I repress. I have had six strokes, so I can not remember any of the details. They simply disappear. I really have to dig deep to remember. It may be that there are questions that I can not answer (P13)*

## The feeling of not being alone

Anxiety and feeling of safety were influenced by the informants being alone at home. For example, the informants considered it as a challenge to be forced to cope with anxiety when discharged from the hospital at night simply by being sent home in a taxi being alone at home.

*Yes, I was comfortable having people around me. The result was the same as at the hospital, but it is of course safer when you are here at home. Anyway, at the hospital, they just send me home (P3)*

Anxiety was expressed as being choked, and not being able to get help.

*I have never been afraid of anything. Now I am suddenly afraid of everything. I do not dare go into the basement because I am terrified that I will be suffocated, it is very uncomfortable. That's actually, well, that's probably what I'm afraid of (P2)*

The informants who had a social network they could rely on support from during an exacerbation of their COPD, expressed this as important in order to cope with the exacerbation and anxiety and in the decision on calling for emergency help.

*[If I had some company] then I do not think I would have been afraid, but I'm alone, and I've said that all along, I'm afraid because I'm alone. My neighbour says that I can just call on her, but still, she is not at home, well she mostly is at home, but then not anyway (P7)*

Two of the patients suggested that they needed a supervised place to stay where they were not alone. They suggested that a sort of nursing facility would be nice during COPD exacerbations.

It was voiced by the informants having someone to be with, when help was needed, could decrease the feeling of anxiety.

*Yes, but heck, back then you were scared every time, and you thought the ambulance took too long to arrive and they probably would not arrive at all. You just sit there and get sicker. I've discovered that I get through it easier because I'm not scared anymore although back then I was scared every time. I thought each time I was going to die, right? (p11)*

## The need for someone to demonstrate professional experience and assistance when falling ill

The informants experienced, that the assessments at the point-of-care treatment did result in a longer stay in informants’ homes. It took additional time to be able to evaluate wherever the informant should be brought to the ED or treatment could be initiated in informants’ homes and subsequently followed up by their GP. The informants expressed this increased time spent at the scene assessing them as being comforting, having professional people in the house who could help them.

*Well, I felt 100% safe when they were here. I was surprised by all the examinations and tests. I had not heard about the project before. (p12)*

It was considered of great importance that the EMTs acted like professionals, radiating experience and confidence when treating the patients. Also, the technical abilities displayed by the EMTs instilled confidence in the patients as it was considered important for the informants to trust that they received the correct evaluation and subsequent treatment.

*I actually felt really safe, I did, I quickly felt comfortable with it because you could clearly see that they had damn good control of it. Also, I knew all along, that we could get to the hospital all the time if it was [necessary] (P12)*

The interviews revealed that to ensure an experience of quality in the assessment and treatment of patients in their own homes, it was important that the ambulance staff showed great safety and experience in the use of the technical equipment and treatment of dyspnoea.

*Oh, I was glad that they helped me breathe again when I just could not [breathe]. It also helped that she, the physician spoke to me, not only to them (EMT’s) but also directly to me [on video screen] (p11)*

Point-of-care diagnostics and treatments of acute COPD exacerbations were considered a qualitative heightening of the prehospital service by the informants and their relatives. The informants experienced great comfort in receiving prompt help from the EMT, not having to explain at first, about their symptoms, COPD, or which help they needed.

*Well, they help immediately. That is the key. They come and help me immediately (P3)*

The informants considered it important that the EMTs were helpful and took the time to inform the patients about the examinations, the assessments, and the treatments. Furthermore, it was considered important that the EMTs took their time to evaluate which actions were important.

EMTs coping with technical problems at patients’ homes were expressed by the patients as being acceptable, because the main issue was, that the EMTs were acting as experienced within coping with symptoms of COPD.

*They were completely professional in their actions. You could not point your finger at anything, their cooperation, it was to a hundred percent (p10)*

## The patient’s everyday relationship with their general practitioner

The informants’ relationship to their GP was considered very important when coping with COPD in exacerbation. After the point-of-care assessment, the informants were referred to follow-up at their GP the next working day. If the informants' GP showed insight regarding the informants’ individual needs and were easy to communicate with (that is, the patient having easy access to the GP and not having to argue too much about the needs), the informants considered it a qualified follow-up to consult the GP. In contrast, if the relationship to the GP was poor, the patient did not trust that the GP could handle their situation.

*Yes, yes, I'm actually mostly at ease [with hospital admission], because then I know that I'm in good hands. They know what it's about, whereas my own doctor does not know much about it, so I'm really most comfortable with admission to the hospital (P4)*

The results showed that it was considered of great importance by the informants that they had confidence that their GP followed up on the home treatment initiated by EMT.

Some of the informants had a perception that their GP did not know anything about lung diseases. This was argued as one of the reasons why they preferred an emergency call, trying to cope with increased symptoms instead of contacting their GP. The informants preferred being admitted to the hospital if they considered their conditions as being life-threatening, expecting the help they needed to be available. Still, the patients trusted the EMT to be able to assess whether admission to the hospital was needed.

Some of the informants that were referred to the GP to follow up on the treatment initiated by the EMT experienced that the day after their in-home treatment, the GP recommended that they were admitted to the hospital. Informants who had a good relationship with their GP used the GP as a partner deciding when and how to manage and treat an exacerbation. If the informant felt confident in the contact with their GP, they would also accept a follow-up after a point-of-care assessment as an acceptable solution.

*But it was nice to have the treatment started right away because otherwise there are a few days where I do not feel very well, where I cannot move around a lot or talk. Yes, I was out of breath, I could not say a whole sentence. (p12)*

The aspect of being able to visit the GP the day after the emergency call, the informants argued as a potential issue. The informants generally were not sure that they were physically able to leave their houses due to continued severe symptoms from the exacerbation.

*Interviewer: Were you seeing the general practitioner the day after the prehospital treatment?*

*No. I was not well enough. Usually, I cannot move around before a few days have passed. I am barely able to use my walker and go to the toilet. With pauses. (P11)*

Informants with firm contact with their GP did not need to physically visit their GP but could simply call the GP and explain in few words how their condition was. This enabled them to get a proper evaluation and follow up on the treatment initiated by the EMT.

## Discussion

We sought to explore the patients’ perspectives on point-of-care treatment at home to patients with COPD in exacerbation through a qualitative research approach. We found that the patients overall accepted the possibility to be treated and released at home. Several conditions were highlighted as considered to have an impact on the feeling of being safe during the examinations performed at home by the EMT. The conduct of everyday life with COPD was considered a condition when informants argued about the meaning of the relation to their GP. This reflects account from another study by Pinnock et al. [22] which showed that patients with COPD regarded COPD as not much an illness, more a way of life condition. The very slowly progressive conditions in COPD symptoms result in an adjustment in their sense of self over the years to fit within their limitations due to COPD symptoms. It results in an acceptance of symptoms with passivity which also has its root in the relation to the GP. This often occurs in a long-term relationship where the GP might share this passive acceptance. In the present study, the informants expressed the onsite examination as a qualitative heightening of the treatment, if they knew that the ensuing contact and follow-up with the GP was easy and could be relied upon without communicative problems. None of the informants expressed that a home visit by their GP was an option. A study by Habraken et al. [23] showed that patients with COPD, despite experiencing numerous symptoms due to COPD, did not regard their limitations caused by the COPD symptoms as abnormal and therefore did not express a need for help. Our present study has shown that a good relationship with the GP was considered important by the informants if they should consider the treat-and-release principle in exacerbations of COPD a viable high-quality solution. Our study also showed that it was considered as important by the informants that the EMT instilled confidence in the patients while treating the COPD symptoms and applying the point-of-care devices. The results from the study by Habraken et al. [23] and Pinnock et al. [22] showed that the inability to express themselves verbally due to shortness of breath in patients with COPD inhibits the communication between patient and caregiver. This previous finding supports the results of our study, as EMTs (and GPs) were considered as qualified by the patients when they displayed experience with the onsite examinations and helping the patients to cope with the COPD symptoms.

Results from a study with evaluation of the diagnostic quality of point of care focused thoracic ultrasound, Pietersen et al. [12], showed that the EMTs should complete an educational program including a theoretical part and a practical part to be able to obtain the images, interpret the images, and can put the findings into context with other investigations. Our study supports this knowledge as our informants expressed the importance of EMT to appear experienced with the technical equipment in order to instill confidence in the patient. Also in the study by Lindelius et al. [24], a higher self-rated patient satisfaction was observed in the group where ultrasound examinations were performed.

The patients expressed that time spent at the scene was comforting because the experienced health professionals were present. Despite the fact that the patients were subjected to a longer period of observation by health care professionals when admitted to the hospital, they preferred to stay at home as being transported to a hospital was considered as stressful.

The patients’ experiences of the admissions to hospital caused by COPD in exacerbation was described in a study by Seamark et al. [25]. In this particular study, the patients also expressed that both the admission and the discharge were considered chaotic. Thus, in our study, the patients were happy to trade a short visit by the ambulance exercising "load-and-go" followed by a short stay at the hospital with a more thorough examination and treatment in their own homes if this could obviate an admission to the hospital. The key point here was the patients’ open expression that they preferred to stay in their homes, if at all possible. We believe that the issue of "professionalism" in this respect is the perceived expertise in handling the point-of-care apparatuses (ultrasonography and blood tests).

Strengths of the study: The qualitative approach as a method used in the present study ensured valid results, as it was possible to extract knowledge on the patients' perspectives on onsite treatment to patients with COPD in exacerbations in emergency calls. Our study elicits how the procedure affected the patients and their relatives and investigated whether the patient perceived the treat-and-release strategy as an acceptable strategy of sufficient quality.

The mayor limitation of the study was that a rather large group of informants was not able to participate in an interview. This was in part because of fatigue due to severe COPD, and in part, because some patients died before the interviews could be performed. Some patients had comorbidities that hindered their participation. Also, a large group of the participants did not remember the emergency call in question either because of cognitive limitations, multiple emergency calls, or severe acute illness when making the emergency call.

A long-time gap between the clinical incident and the interview with some informants could have caused a recall bias. The informants that could not recall the visits in this study all had well-known memory problems. The gap of time from the incident to the interviews also resulted in some informants having had new incidents with emergency calls. However, we consider this a strength in the interviews, because the difference between a regular emergency call and project visits appeared more clearly to the informants.

## Conclusion

From a patient perspective, it is considered feasible for prehospital EMTs to perform point-of-care ultrasound and laboratory tests in patients with COPD in acute exacerbations. The patients expressed that the perceived experience of the EMTs was considered important, not only concerning the handling of the point-of-care equipment but also in helping patients to cope with the symptoms of COPD (dyspnoea, anxiety, and the difficulties in speaking due to shortness of breath). The patient’s perspective was that the time spent on examinations at the scene was considered as instilling confidence by the presence of experienced EMTs. Also not being alone was argued as important. Furthermore, avoiding a short admission to the hospital was considered an important issue by the informants. The follow-up by GP was considered different among the informants. This was influenced by the quality of the informants’ relationships with their GPs and whether GP’s knowledge of COPD was perceived as high by the informants. Finally, the informants regarded it as comfortable to get a follow-up at the GP. If the informants regarded their GP as not having enough knowledge about COPD, however, they preferred the treatment initiated by the EMT or the hospital.

## Data Availability

The dataset generated and analysed during the current study is not publicly available due to ethical reasons the efforts to protect the participants’ identity.

## Abbreviations

COPD: Chronic obstructive pulmonary disease
GP: General practitioner
EMT: Emergency medical technician
ED: Emergency department
